# Parthenolide and DMAPT induce cell death in primitive CML cells through reactive oxygen species

**DOI:** 10.1111/jcmm.13755

**Published:** 2018-08-05

**Authors:** Gabriela Flores‐Lopez, Dafne Moreno‐Lorenzana, Manuel Ayala‐Sanchez, Socrates Aviles‐Vazquez, Hector Torres‐Martinez, Peter A. Crooks, Monica L. Guzman, Hector Mayani, Antonieta Chávez‐González

**Affiliations:** ^1^ Leukemic Stem Cells Lab Oncology Research Unit Mexican Institute of Social Security Oncology Hospital “Siglo XXI” National Medical Center Mexico City Mexico; ^2^ Hematology Department & BMT Unit Medical Specialties Hospital “La Raza” Medical Center Mexican Institute of Social Security Mexico City Mexico; ^3^ Department of Hip Surgery Mexican Institute of Social Security “Villa Coapa” General Hospital Mexico City Mexico; ^4^ Division of Hematology/Medical Oncology Department of Medicine Weill Medical College of Cornell University New York NY USA; ^5^ Hematopoietic Stem Cells Lab Oncology Research Unit Mexican Institute of Social Security Oncology Hospital “Siglo XXI” National Medical Center Mexico City Mexico; ^6^ Department of Pharmaceutical Sciences College of Pharmacy University of Arkansas for Medical Sciences Little Rock AR USA

**Keywords:** CML, leukaemic stem cell, parthenolide, ROS

## Abstract

Tyrosine kinase inhibitors (TKI) have become a first‐line treatment for chronic myeloid leuakemia (CML). TKIs efficiently target bulk CML cells; however, they are unable to eliminate the leukaemic stem cell (LSC) population that causes resistance and relapse in CML patients. In this study, we assessed the effects of parthenolide (PTL) and dimethyl amino parthenolide (DMAPT), two potent inhibitors of LSCs in acute myeloid leukaemia (AML), on CML bulk and CML primitive (CD34^+^lin^−^) cells. We found that both agents induced cell death in CML, while having little effect on the equivalent normal hematopoietic cells. PTL and DMAPT caused an increase in reactive oxygen species (ROS) levels and inhibited NF‐κB activation. PTL and DMAPT inhibited cell proliferation and induced cell cycle arrest in G_0_ and G_2_ phases. Furthermore, we found cell cycle inhibition to correlate with down‐regulation of cyclin D1 and cyclin A. In summary, our study shows that PTL and DMAPT have a strong inhibitory effect on CML cells. Given that cell cycle arrest was not dependent on ROS induction, we speculate that this effect could be a direct consequence of NF‐κB inhibition and if this mechanism was to be evaded, PTL and DMAPT induced cell death would be potentiated.

## 1 INTRODUCTION

Chronic myeloid leukaemia (CML) is a clonal myeloproliferative malignancy originating in a leukaemia stem cell population (LSC).^1^ CML cells express the Philadelphia chromosome (Ph), the product of the balanced translocation t(9;22)(q34;q11) of the long arms of chromosomes 9 and 22,^2^ giving rise to the oncoprotein p210 Bcr‐Abl.^3^, ^4^ Bcr‐Abl is a tyrosine kinase with constitutive activity that drives downstream activation of various signalling pathways that mimic growth factor stimulation, giving cells the ability to evade apoptosis, increase proliferation, differentiation and alter cell adhesion. Together, these features characterize CML cells and drive the progression of CML.^5^

Diagnosis in 90% of cases occurs at chronic phase (CP), where patients have abnormal cell counts but myeloid cells retain differentiation ability.^6^ If not treated, patients progress to an accelerated phase and finally to blast crisis (BC),^6^ which is invariably fatal. Knowledge of the molecular biology of CML has provided the development of tyrosine kinase inhibitors (TKIs), able to directly target Bcr‐Abl, and inhibit its downstream activity, offering patients in CP the possibility of achieving remission and prolong survival. TKI's, however, are not able to target LSCs, a rare, quiescent, self‐renewing cell population that initiates and sustains CML.^6^ Recently, Nieborowska‐Skorska and colleagues have reported that LSC from CML patients present high levels of ROS compared to normal primitive cells.^7^ This has been related to defects in mitochondria of CML cells, which cause the accumulation of ROS, driving genomic instability and acquired resistance to TKIs.^7^ This raises the interest in seeking targets other than Bcr‐Abl that may be able to eliminate LSC, which are not eliminated with current TKI therapies.

Medicinal plants with anti‐inflammatory properties are rich reservoirs of bioactive compounds.^8^ Parthenolide (PTL) is a sesquiterpene lactone extracted from the plant *Tanacetum parthenium*, used by folk medicine for anti‐inflammatory purposes, arthritis, migraine, as an anticoagulant, and a digestive aid, for centuries.^9^ Guzman and collaborators have reported that PTL is able to target activity against LSC in AML.^10^ To improve the pharmacological properties of PTL, an orally bioavailable analogue was generated, dimethyl amino parthenolide (DMAPT) that showed similar activity to PTL in AML LSC^11^; nevertheless, the effect on CML cells, including both primitive primary cells, as well as CML cell lines, is unknown. The mechanism of action of PTL has been reported to rely on a number of pathways, two of which are the inhibition of NF‐κB via the alkylation of critical cysteine residues^12^ and the induction of ROS by affecting the glutathione system.^13^

We present evidence that PTL and DMAPT are able to abrogate bulk CML cells and a primitive CML population (CD34^+^lin^−^) by a mechanism dependent on ROS induction and NF‐κB pathway inhibition. The effect on ROS levels is transitory, reaching a peak at 1 hour of treatment.

In addition, we observed the up‐regulation of HMOX‐1 which is activated by Nrf2 transcription factor, described as an integrator of cellular stress signals^14^ and promotes survival of a fraction of CML cells. These cells demonstrate a cell cycle arrest in G2/M and G0 cell cycle phase, which was accompanied by changes in Cyclin D and A, and down‐regulation of CDK2.

## 2 METHODS

### 2.1 Chemicals and reagents

PTL and DMAPT were kindly provided by Drs. Cesar Compadre and Peter Crooks, respectively (University of Arkansas for Medical Sciences). Both agents were kept at ‐20 C at 40 mmol/L in DMSO.

### 2.2 Cell lines and primary samples

CML and AML cell lines were purchased from ATCC and authenticated by STR repeats (Biosynthesis, Lewisville TX). The presence of Bcr‐Abl transcript was verified for Meg‐01, K562, Kasumi‐4 and KCL‐22 cells. Cells were cultured in RPMI 1640 culture medium supplemented with FBS (10%) and Penicillin‐Streptomycin (PS) (1%). HL60 cell lines, were cultured in IMDM at 20% of FBS and 1% PS. Cell lines were cultured at a density of 3 × 10^5^ per mL. Primary samples from normal bone marrow (NBM) (n = 10) and CML Chronic Phase before TKI treatment (n = 10) were collected according to institutional guidelines, including written informed consent from each donor. All procedures were approved by the Ethics and Scientific Committee at IMSS (Protocol # R‐2012‐785‐052).

### 2.3 Stem and progenitor cell enrichment

Mononuclear cells (MNC) from primary samples were isolated using Ficoll‐Paque Plus (Pharmacia Biotech, Uppsala, Sweden). Primary CD34^+^lin^−^ were enriched and cultured from MNC after negative selection as previously described.^15^

### 2.4 Cell cultures for cell viability and colony forming cells (CFC) assays

Cell viability was assessed by trypan blue exclusion. Cells were cultured in the presence or absence of PTL or DMAPT, after 24 or 48 hours, cells were harvested and counted with trypan blue at 0.4% in a Neubauer chamber. For CFC assays, 5000 viable CD34^+^lin^−^ cells were cultured for 14 days in methylcellulose‐based semisolid medium (Methocult Classic with cytokine, STI) after 24 hours of PTL or DMAPT treatment. Colonies were evaluated using an inverted microscope.

### 2.5 Cell division and cell death assays

Cell division tracking was performed with carboxy‐fluorescein diacetate succinidmidyl diester (CFSE). Cells were stained with 1 mmol/L CFSE and cultured in the presence or absence of PTL or DMAPT. After 24 hours, cells were harvested, washed and analysed by flow cytometry. Cell death was determined by staining with YoPro‐1 and 7AAD, or Annexin‐V/7AAD apoptosis detection kit (BD Biosciences) and flow cytometry analysis. The apoptotic index was determined by calculating the fold change in apoptotic cells (Yopro‐1/7AAD or Annexin‐V/7AAD positive) in PTL or DMAPT treated cells compared to the death cells in Untreated groups (considered as 1). Caspase dependent cell death was determined by pre‐treatment of cells with Z‐vad (100μmol/L) for 2 hours previous to PTL and DMAPT treatment. Cell viability was determined after 24 hours and caspase 3 activation by detection of the active form of caspase 3 (C92‐605, BD Biosciences, Mountain View, CA) by flow cytometry.

### 2.6 Reactive oxygen species

ROS levels were assessed by dichlorofluorescein diacetate (DCFDA) (Sigma), or costained with Cell Rox deep red and Mitosox (Thermo Fisher). Cells were stained and incubated at 37°C for 30 minutes, according to manufacturer's instructions, and exposed to PTL or DMAPT for 1 or 6 hours.

### 2.7 Gene expression assay

Cells from cell lines or CD34^+^lin^−^ from primary samples were cultured for 6 hours with or without PTL or DMAPT. Cells were harvested and RNA was extracted using RNA‐easy Kit (Qiagen, Hilden, Germany), according to manufacturer's instructions. Quantitative RT‐PCR was performed using Taqman RNA to Ct 1 step assay (Applied Biosystems, Foster City, CA), and Taqman assays to evaluate the expression of the following genes: NFKB1 (Hs00765730_m1), HMOX‐1 (Hs01110250_m1), PLAU (Hs01547054_m1) and GAPDH (Hs02758991_g1).

### 2.8 Immunofluorescence

K562 cells were cultured for 6 hours with PTL or DMAPT, cells were pelleted and 100‐200 thousand were spread in slides and fixed in Methanol at −20°C. Cells were permeabilized with Tween 20 at 0.1% in PBS at room temperature and stained for anti‐p65 antibody (c‐20, Santa Cruz Biotechnology, Santa Cruz, CA). Slides were washed and stained with secondary anti Rabbit IgG Alexa Fluor 488 conjugate (Thermo Fisher Scientific). Samples were mounted using Fluoromount‐G with DAPI and images were acquired using an EVOS microscope (Life Technologies, Grand Island, NY, USA).

### 2.9 Immunoblots

Cells were cultured for 6 hours with or without PTL or DMAPT, cells were counted, washed and pellet was resuspended and lysed at 20 million/mL in lysis buffer (10 mmol/L Tris, Ph 7.5, 50 mmol/L NaCl, 30 mmol/L Na_4_P_2_O_7_, 50 mmol/L NaF, 5 μmol/L ZnCl2, 1% Triton X‐100), with protease and phosphatase inhibitors: 1 mmol/L Phenylmethanesulfonyl fluoride (Sigma‐Aldrich), 1X Protease Inhibitor Cocktail (Merck‐Millipore) and 20 μmol/L Na3VO4 (Sigma‐Aldrich). Proteins were preserved at−80°C and extract from 250,000 cells was loaded in NU‐PAGE 7% pre‐cast gels (Thermo Fisher Scientific). Gels were transferred to Polyvinylidene fluoride membrane (Immobilon^®^‐FL Membrane, Merck‐Millipore), blocked with 5% Bovine serum albumin, incubated with anti‐phospho p65 (Cell Signaling ser536 93H1 rabbit mAb), anti‐p65 (Santa Cruz C‐20) and anti‐beta actin (Sigma‐Aldrich) in 1% Bovine serum albumin, and blotted with secondary IrDye 800CW goat antimouse IgG (Li‐COR Biosciences) or IrDye 680RD goat anti‐rabbit IgG (Li‐COR Biosciences), proteins were detected using a Li‐COR scanner (Biosciences).

### 2.10 Cell Cycle and cell cycle regulator detection

Cells were incubated in the absence or presence of PTL or DMAPT for 48 hours and fixed with 70% ethanol at −20°C. Cells were permeabilized using 0.2% Triton × 100 in PBS solution and stained at 4°C with anti Ki67‐FITC (BD Biosciences) in 10% FBS. To evaluate CDKIs content, cells were incubated overnight with anti‐p21 (Santa Cruz), anti‐CDK2 (78B2, Cell Signaling Technology), anti‐cyclin D1 (92G2, Cell Signaling Technology), anti‐cyclin A2 (BF683, Cell Signaling Technology), 7‐AAD (559925, BD Biosciences) and secondary antibodies Alexa 488 antimouse IgG (4408, Cell Signaling Technology) and Alexa 647 anti rabbit IgG (4414, Cell Signaling Technology) analysed by flow cytometry.

### 2.11 Cytometry Analysis and Statistical Analiysis

All Flow cytometry assays were done in a BD FACS Verse, BD FACS CANTO or BD FACS LSR II. Flow cytometer data was analysed with Flow‐jo Software.

All data is expressed as Mean ± SEM.

Statistical analysis for each experiment is specified in each figure. Graph and statistical analysis was done using Graphpad Software.

## 3 RESULTS

### 3.1 PTL and DMAPT decrease viability of CML bulk and progenitor cells

**Figure 1:**
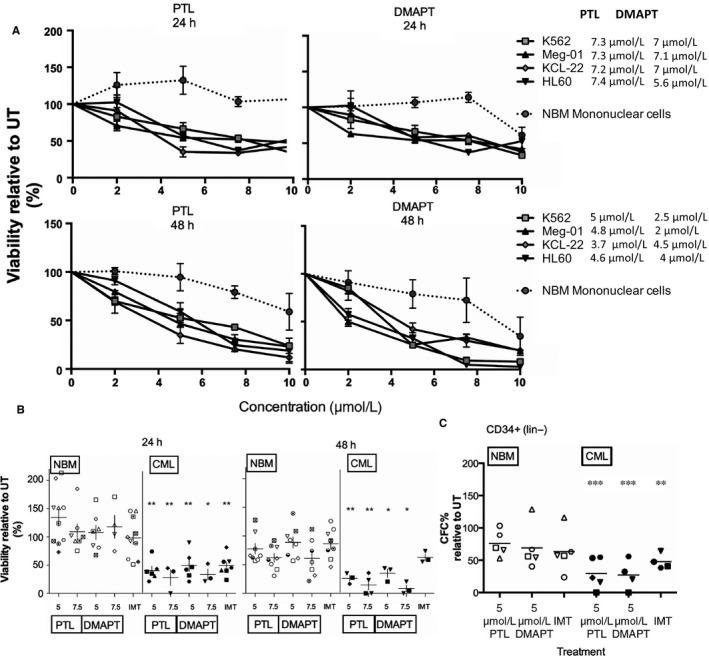
PTL and DMAPT reduce viability of CML bulk and primitive cells over NBM cells**.** Leukaemic cell lines and primary samples from CML patients and normal bone marrow were cultured for 24 or 48 h at different concentrations of PTL and DMAPT. Results in (A) correspond to the viability of Normal Mononuclear Cells (NBM), K562, KCL‐22 and MEG01 CML cell lines and HL60 AML cell line (used as a positive control). The IC50 is shown in the right side of the graph. B, represents the viability of MNC from normal (NBM) and CML primary samples after at 5 and 7.5 μmol/L of PTL and DMAPT for 24 and 48 h of culture, symbol represents significance between NBM and CML groups, determined by Student's *t‐*test. C, indicates the per cent of CFC from normal (NBM) and CML CD34^+^lin^−^cells exposed to PTL and DMAPT in liquid culture by 24 h and then sub‐cultured in methylcellulose for 14 days, stars represent significance with UT group, determined using Dunnet's multiple comparison test. *P < .05 ** *P* < .01, *** *P* < .001

To assess the effect of PTL and DMAPT on viability, three CML cell lines (K562, Meg‐01, and KCL‐22) were exposed to increasing concentrations of the compounds for 24 and 48 hours. As a normal control, MNC from NBM were used, as well as AML cell line HL60, previously reported as PTL sensitive.^16^ We found a significant decrease in CML cell line viability by trypan blue (Figure 1A), the LC50 we observed (7.5 μmol/L at 24 hours, and 5 μmol/L at 48 hours) were similar to the LC50 reported for other leukaemias^10^ (Figure 1A), importantly this was not observed in MNC from NBM cells, we did however observe a significant reduction in NBM cell viability at the highest concentration of DMAPT (20 μmol/L). Interestingly, an increase in the number of viable cells was observed in MNC from NBM exposed to low concentrations of PTL (2 μmol/L). This is in concordance with a previous report by Li‐Weber and colleagues^17^ in which PTL at low concentrations (2 μmol/L) induced apoptotic inhibition on T cells. This may also be because of an increase in ROS levels, as an increase in proliferation has been reported by Day and Susuki for fibroblasts exposed to low levels of ROS.^18^

After determining the LC50 for CML cell lines, we chose to treat primary MNC from CML and NBM with 5 and 7.5 μmol/L PTL and DMAPT, for 24 and 48 hours. The TKI imatinib (IMT, at 2.5 μmol/L) was used to compare effect on primitive and bulk CML cells to parthenolide and DMAPT treatment. As shown in Figure 1B, PTL and DMAPT treated MNC from CML showed a reduction in cell viability of 40% and 30% of live cells at 24 and of 30% and 20% at 48 hours, compared with their normal counterparts that show a viability of 100% and 70% with the same treatments. This indicates a window of treatment at 5 μmol/L and below 10 μmol/L, where CML cells lose viability while NBM cells are spared. In trying to determine if sesquiterpene lactones affected a more primitive population, CD34^+^lin^−^ cells were cultured for 24 hours with or without 5 μmol/L PTL or DMAPT and cultured in methylcellulose for CFC assessment. CFC in CML cultures were significantly reduced in PTL and DMAPT to 30% compared to untreated, while imatinib treated CFC decreased to 50% (Figure 1C). Interestingly, cells from NBM did not show a significant decrease in CFC, suggesting a possible selectivity in the leukaemic population that could be targeted by PTL and DMAPT.

### 3.2 PTL and DMAPT induce cell death in CML cells

**Figure 2:**
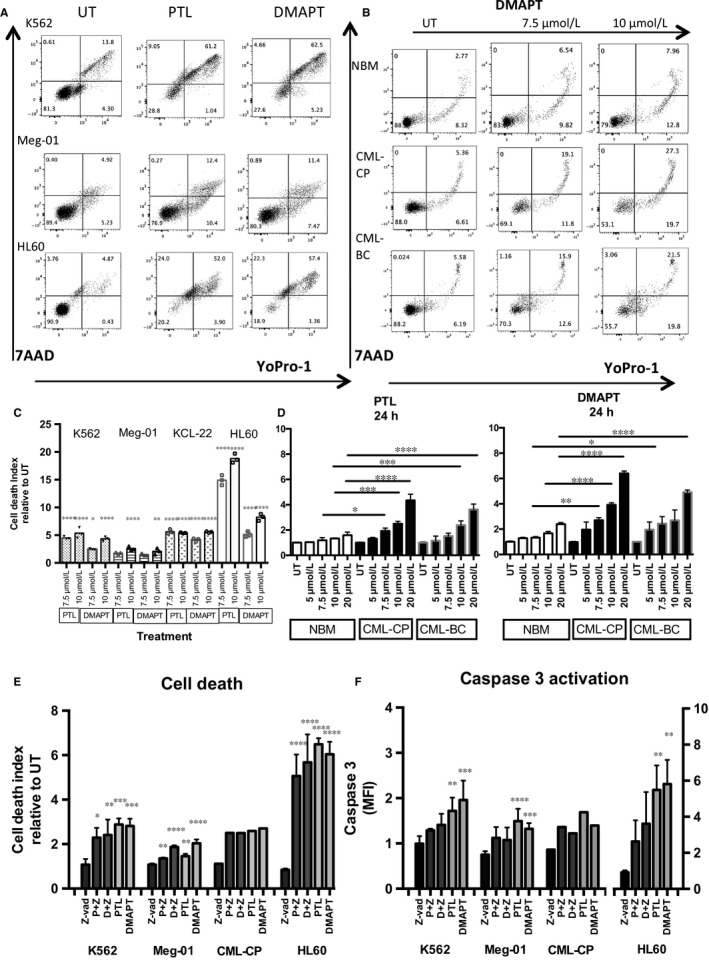
PTL and DMAPT induce cell death in CML cells. Leukaemic cell lines and primary samples from CML patients and normal bone marrow were cultured for 24 h at different concentrations of PTL and DMAPT and the cell death index was analysed. A, Shows flow cytometry plots from K562 and Meg‐01 CML and HL60 AML cell line (used as a positive control). B**,** Shows representative plots from 3 primary samples (Normal Bone Marrow and CML from a chronic phase and a blast crisis patient). ex in Untreated conditions or exposed to 7.5 or 10 μmol/L of DMAPT. Lower and left arrows indicate fluorochrome stain, Upper arrow indicates DMAPT concentration. C**,** Represents the cell death index detected in K562, Meg‐01, KCL‐22 and HL60 cell lines and D Indicates the average of 3 replicates with primary samples from normal bone marrow (NBM), CML Chronic Phase (CML‐CP) and CML Blast Crisis (CML‐BC) mononuclear cells. E, Represents the cell death index increase (YoPro‐1 and Propidium Iodide) in cells from cell lines and a CD34+ enriched CML‐CP primary sample, pre‐treated with the pan‐caspase inhibitor Z‐vad and PTL or DMAPT. F, Shows the activation of Caspase‐3 after pre‐treatment with Z‐vad and treatment with PTL or DMAPT for 24 h. Cell death index was determined by dividing the frequency of dead cells in PTL or DMAPT treated cells by the frequency of dead cells detected in untreated group, (considered as 1). Significance between cell death index in UT and treated in cell line experiments was determined by Dunnet's multiple comparison test and between NBM treated and CML treated cells was determined by Tukey's multiple comparison test. *P < .05 **P < .01, ***P < .001, ****P < .0001

To determine if viability loss was related to apoptosis, CML cell lines K562, KCL‐22 and Meg‐01 were treated with 7.5 and 10 μmol/L PTL or DMAPT, and showed an increase in cell death, compared with untreated (Figure 2A,B). The AML cell line HL60 was used as control and showed the highest death level.

To observe this effect in primary samples, MNC from NBM and CML patients (CP and BC), were exposed to PTL and DMAPT. Figure 2D shows a representative result on the progressive induction of cell death at increasing doses of DMAPT. While DMAPT induced a fold increase of 6 and 5 in cell death at its highest concentration, only induced a 2.8‐fold increase in normal cells, the death index in CML cells was always higher than the one of NBM cells.

With the goal to determine if the cell death in CML cells could be related with Caspase 3 activation, we cultured CML cells in the presence of the pan‐caspase inhibitor Z‐VAD. Our results indicate that Caspase 3 (Figure 2F) is activated in response to PTL or DMAPT in CML cells, while cells pre‐treated with Z‐vad (100 μmol/L) have a decreased Caspase‐3 activation, this decrease does not translate in a decrease in cell death (Figure 2E), suggesting that cell death associated with PTL and DMAPT treatment is mostly caspase independent, as has been reported for other anticancer agents in leukaemia cells,^19^ as well as has been reported for osteosarcoma cells where PTL induces autophagy and mitophagy associated with the increase in reactive oxygen species.^20^ Interestingly, in the case of HL60, AML cell line the cell death index appear to be associated with caspase 3 activation (Figure 2D).

### 3.3 PTL and DMAPT induce Reactive Oxygen Species (ROS) in CML Cells

**Figure 3:**
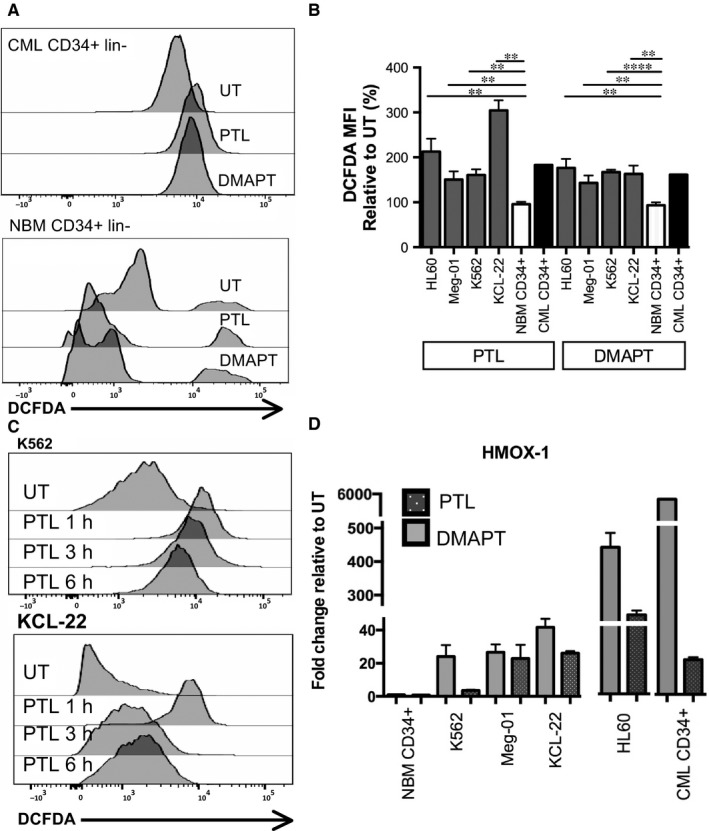
PTL and DMAPT induce Reactive Oxygen Species and activate Haemoxigenase‐1 transcription. Leukaemic cell lines and progenitor cells from CML patients and NBM were cultured in the presence or absence of PTL and DMAPT. ROS levels were quantified with DCFDA stain by flow cytometry. A representative histogram from normal and CML CD34^+^lin^−^ cells treated for 1 h is shown in A**.** B**,** represents the ROS levels in CML and NBM cells, DCFDA MFI levels were normalized to untreated cells to evaluate ROS level changes with treatment. C**,** represents changes in ROS levels in CML cell lines at 1, 3 and 6 h of exposure to PTL. Haemoxigenase‐1 transcription was determined by qPCR in primary CML and normal bone marrow CD34^+^lin^−^ cells, as well as CML (K562, Meg‐01, KCL‐22) and AML (HL60) cell lines, exposed for 6 h to PTL or DMAPT. Results represent the fold change relative to basal transcription without treatment, **D**. Significance between DCFDA fold change in NBM CD34+ and leukaemia cell lines was determined using an Unpaired Student's *T* test. **P < .01 and ****P < .0001

Previous reports demonstrated that ROS levels are increased in CML primitive cells.^7^ Considering that PTL and DMAPT have been reported as ROS inducers in different cancer models,^21^ we assessed this effect in CML cells. At 1 hour of PTL or DMAPT treatment CML cells from cell lines and CD34^+^lin^−^ CML‐CP showed an increase in DCFDA MFI, compared to untreated cells, indicating an increase in ROS levels, importantly primitive cells from NBM did not show an increase in ROS levels. Furthermore, basal ROS levels were higher in CML primitive cells (Figure 3B), such as has been previously reported.^7^ Also, NBM cells responded differently to treatment, ROS levels in CML CD34^+^lin^−^ were increased while in the normal counterpart were reduced, suggesting a higher resilience to oxidant stress and an antioxidant mechanism in the normal population. The initial evaluation of ROS, showed the increase to be transitory, after 3‐6 hours of treatment K562 and KCL‐22 cells decreased ROS levels from the initial peak at 1 hour, suggesting cells activate defense mechanisms (Figure 3C). Thus, we looked at Hemoxigenase‐1 transcription at 6 hours of PTL or DMAPT treatment by q‐PCR and observed that all CML cell lines (K562, MEG01, KCL‐22 and Kasumi‐4) showed up‐regulation of HMOX‐1 transcription, the effect was significantly higher in CD34^+^lin^−^ CML‐CP samples. Interestingly, the effect was not observed in the normal cell counterpart in response to both agents (Figure 3D). This effect could be attributed to the lack of ROS induction, which would not activate the Nrf2 pathway responsible for HMOX‐1 transcription.

### 3.4 PTL and DMAPT induced cell death is ROS dependent

**Figure 4:**
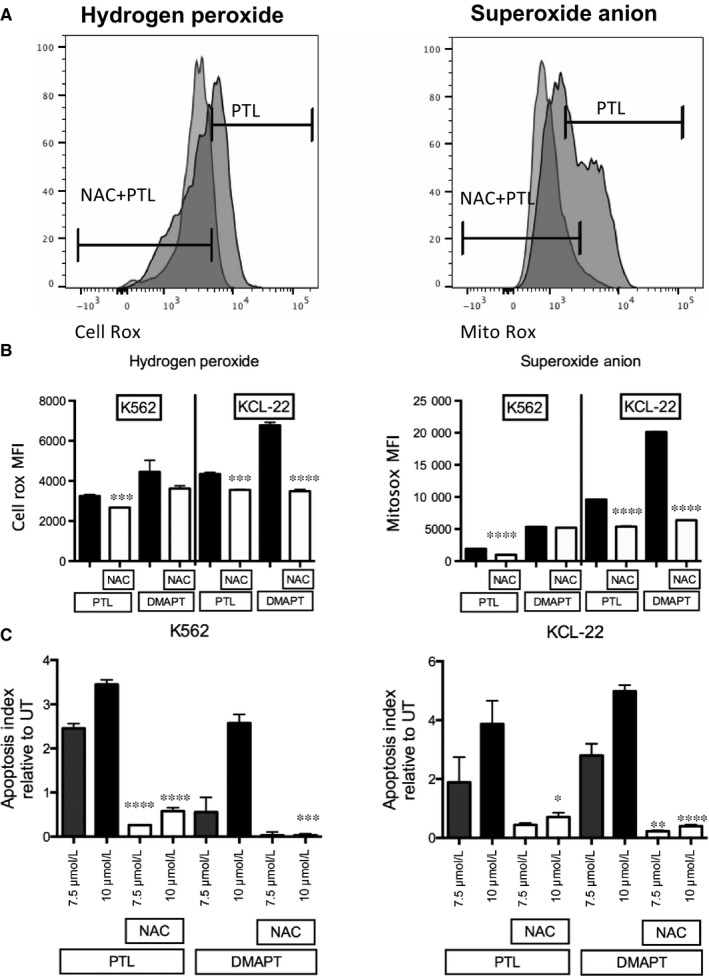
Cell death induced by PTL and DMAPT depends of ROS induction. K562 and KCL‐22 cell lines were pre‐treated with N‐acetyl cysteine (NAC) for 1 h and subsequent treatment with PTL or DMAPT at 7.5 μmol/L or 10 μmol/L, ROS levels and cell death were analysed by flow cytometry. A**,** Shows a representative histogram of cells pre‐treated with or without NAC and treated afterwards with PTL. B, represents MFI levels of specific ROS (Hydrogen Peroxide or Superoxide Anion) in NAC pre‐treated cells compared to those treated with PTL or DMAPT for 6 h (C) indicates the cell death index after 24 h of treatment with PTL or DMAPT in NAC pre‐treated cells compared to those treated only with PTL or DMAPT. Significance between treated cells and control in MFI of ROS levels and cell death index was determined using an unpaired Student *t*‐test. *P < .05 **P < .01, ***P < .001, ****P < .0001

We next analysed whether cell death in CML cells is ROS dependent. K562 and KCL‐22 cell lines were pre‐treated with the ROS scavenger NAC at 15 mmol/L, for 1 hour; the cells were then cultured in fresh media with or without PTL and DMAPT at 7.5 or 10 μmol/L. At 6 hours of treatment, half of the cells were harvested for ROS detection and stained with CellROX‐APC (hydrogen peroxide indicator) and Mitosox Red (superoxide anion indicator). NAC pre‐treatment reduced ROS induced by PTL or DMAPT, and data suggest that the major effect on ROS is mediated by superoxide anion (Figure 4A). At 24 hours of treatment, cell death was determined. NAC pre‐treatment was able to rescue cells from death induced by PTL or DMAPT (Figure 4C), indicating that ROS induction is a key factor in cell death induced by these agents (Figure 4B).

### 3.5 PTL and DMAPT inhibit NF‐κB pathway in CML cells

**Figure 5:**
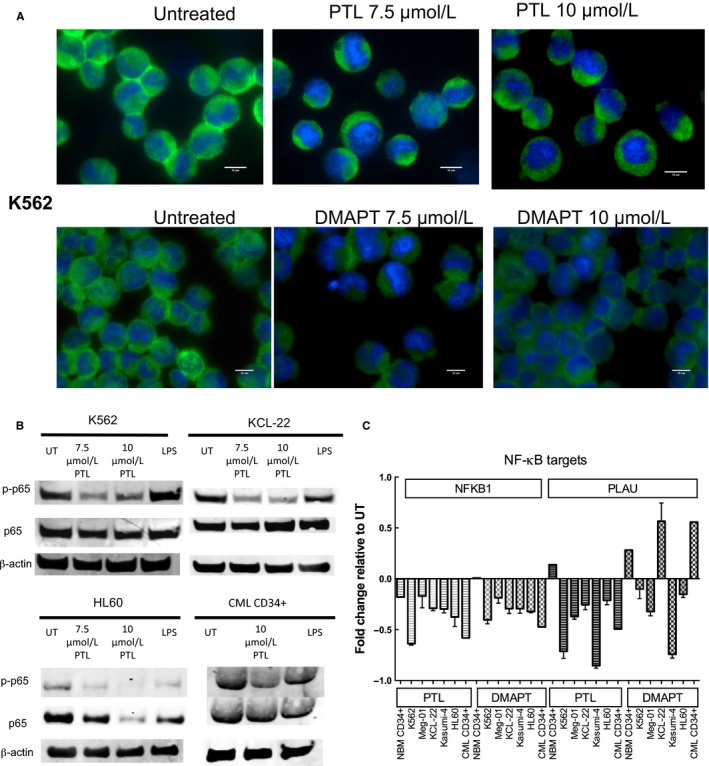
PTL and DMAPT treatment correlates with NF‐κB inhibition in CML cells. Leukaemic cell lines and primary CD34^+^lin^−^ cells from CML patients and normal bone marrows were cultured in the absence or presence of 7.5 and 10 μmol/L PTL or DMAPT. Results in (A) represent an immunofluorescence analysis for total p65 and nuclear stain with DAPI in K562 cell line. B, correspond to a representative Western Blot for phospho p65 and p65 total, using LPS as a positive control of NF‐κB induction and beta‐actin as a loading control. C, Represents analysis of NF‐κB transcription targets (NFKB1 and PLAU) in cells treated with PTL or DMAPT for 6 h. Scale bar: 10 μm

PTL and DMAPT have been reported as NF‐κB pathway inhibitors, although the molecular mechanisms have not been completely elucidated, a report by García Piñeres and colleagues suggest that PTL blocks NF‐κB pathway by alkylation of cysteine residues in p65.^12^ Other authors have reported that the mechanism relies on an upstream inhibition of the NF‐κB pathway, by inhibiting the activity of IKK.^10^ Furthermore, PTL activity has been reported to rely on the alkylation capacity of critical serine residues.^12^ To verify if PTL and DMAPT were able to inhibit NF‐κB, K562 cells were treated for 6 hours with PTL or DMAPT at 7.5 and 10 μmol/L and p65 total protein localization was assessed by immunofluorescence (Figure 5A). K562 cells showed an overall decrease in p65 nuclear localization, suggesting pathway inhibition. To verify that nuclear localization inhibition was due to inhibition of p65 phosphorylation, CML cell lines K562 and KCL‐22, the AML HL60 cell line, and primitive cells from a primary CML‐CP sample were treated with PTL at 7.5 and 10 μmol/L, for 6 hours. Total p65 protein levels remained unchanged after treatment, while phospho‐p65 levels diminished with PTL treatments in both CML cell lines and in a primitive population from a CML patient, indicating an inhibition of the NF‐κB canonical pathway (Figure 5B). Furthermore, to verify the functional status of NF‐κB, transcriptional activity of the pathway was evaluated in CML cell lines (K562, Meg‐01, KCL‐22, Kasumi‐4), AML cell line HL60 and primary CD34^+^lin^−^ cells from CML and NBM samples, according to Guzman and collaborators.^22^ Cells were cultured for 6 hours in the absence or presence of PTL or DMAPT at 7.5 μmol/L. RNA from all cells was extracted and the relative expression of NFKB1 (p50), PLAU (plasminogen activator, urokinase) and GAPDH (endogenous control) were determined. Fold change in expression was calculated as the expression relative to UT, using the 2‐ΔΔCT method by Livak and Schmittgen.^23^ NFKB1 levels consistently decreased with PTL or DMAPT treatment; interestingly no change was observed in NFKB1 levels in NBM primitive cells with DMAPT (Figure 5C). A small down‐regulation was observed in NBM primitive cells with PTL. In the case of PLAU, there was a reduction in NFKB1 levels in all leukaemic cells but not in normal counterpart, in contrast PLAU appear to be differentially regulated in each one of the cell types in response to DMAPT (Figure 5C) and this effect could be related with another transcriptional complex or microRNA expression as have been reported in solid tumours.^24^, ^25^

### 3.6 PTL and DMAPT inhibit proliferation and arrest Cell Cycle of CML cells

**Figure 6:**
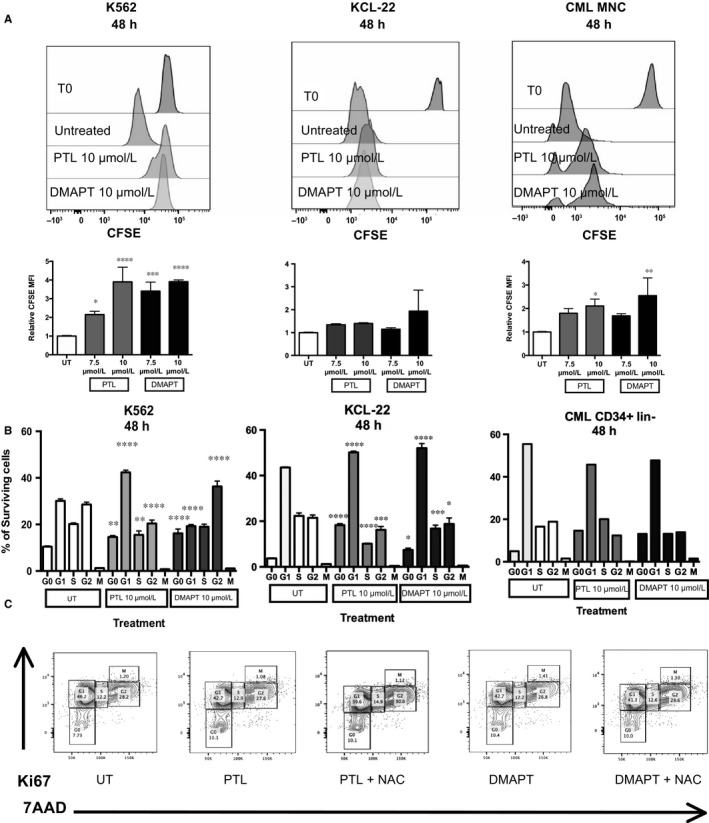
PTL and DMAPT delay cell proliferation and induce an arrest in G0 cell cycle phase in a mechanism independent of ROS induction. Leukaemic cell lines were cultured in the absence or presence of 7.5 and 10 μmol/L to PTL and DMAPT for 48 h, surviving cells were obtained by ficoll‐paque and selected as 7AAD negative cells. CFSE content and cell cycle status was analysed by flow cytometry. Results in (A) show a representative histogram of CFSE level after PTL or DMAPT treatment as well as MFI before treatment. The MFI values from 3 experiments are plotted below. (B) indicates a representative plot of cell cycle distribution and the average of cell cycle phases. In the case of C, the cells were pre‐treated with NAC for 1 h and then cultured with PTL and DMAPT for 48 h. The result shows a representative histogram of tree different replicates. Significance between UT and treated cells in proliferation assay was determined using an unpaired Student *t*‐test. Significance between Cell cycle phase was determined using Tukey's multiple comparison test. *P < .05 **P < .01, ***P < .001, ****P < .0001

Current objectives in CML therapy include the eradication of LSC remaining after TKI therapy and that cause relapse in patients.^26^ Although TKI treatments are very effective targeting Bcr‐Abl activation, the quiescent leukaemic pool has been reported as not sensitive to this inhibition.^27^ Recently, our group reported that the G0/G1 phase fraction increases in CML cells after being treated with TKIs, and that this is dependent on nuclear localization of CDK inhibitors p21 and p18.^15^ NF‐κB has also been reported as a regulator of cell cycle, and it is therefore relevant to all new therapeutic strategies. A first approach to analyse this was to verify the effects of PTL and DMAPT on cell proliferation. K562, KCL‐22 and primary CML MNC were pre‐stained with CFSE and cultured in the presence or absence of sesquiterpene lactones (at 7.5 or 10 μmol/L) for 48 hours, and were stained with 7AAD to select for surviving cells and analysed by flow cytometry. After 48 hours of treatment, a tendency towards proliferation inhibition in all cell lines was observed. K562 surviving cells showed a significant increase in CFSE fluorescence when treated with PTL or DMAPT (Figure 6A,B).

These observations suggested an effect on cell cycle. Accordingly, we treated CML cells (K562, Kcl22, CML primitive cells) with 10 μmol/L PTL or DMAPT for 48 hours and assessed cell cycle status by flow cytometry. All CML cells showed a consistent increase in G0 cell phase, K562 cell line also showed an increase in the G2 phase, with a consistent decrease in S phase in all cells (Figure 6B,C). The consistent reduction in the S phase fraction could indicate a loss of commitment to DNA synthesis due to NF‐κB inhibition, which has been reported to regulate cyclin D expression, a crucial component in DNA synthesis commitment.

In an effort to understand the role of ROS on cell cycle arrest, the K562 cell line was pre‐treated with NAC (a ROS scavenger) for 1 hour at 15 mmol/L. Interestingly, cells pre‐treated with NAC indeed changed cell cycle status when they were treated with PTL or DMAPT, indicating that the cell cycle effect is not dependent on ROS increase.

### 3.7 Cell cycle arrest by PTL and DMAPT is related to cyclin down‐regulation

**Figure 7:**
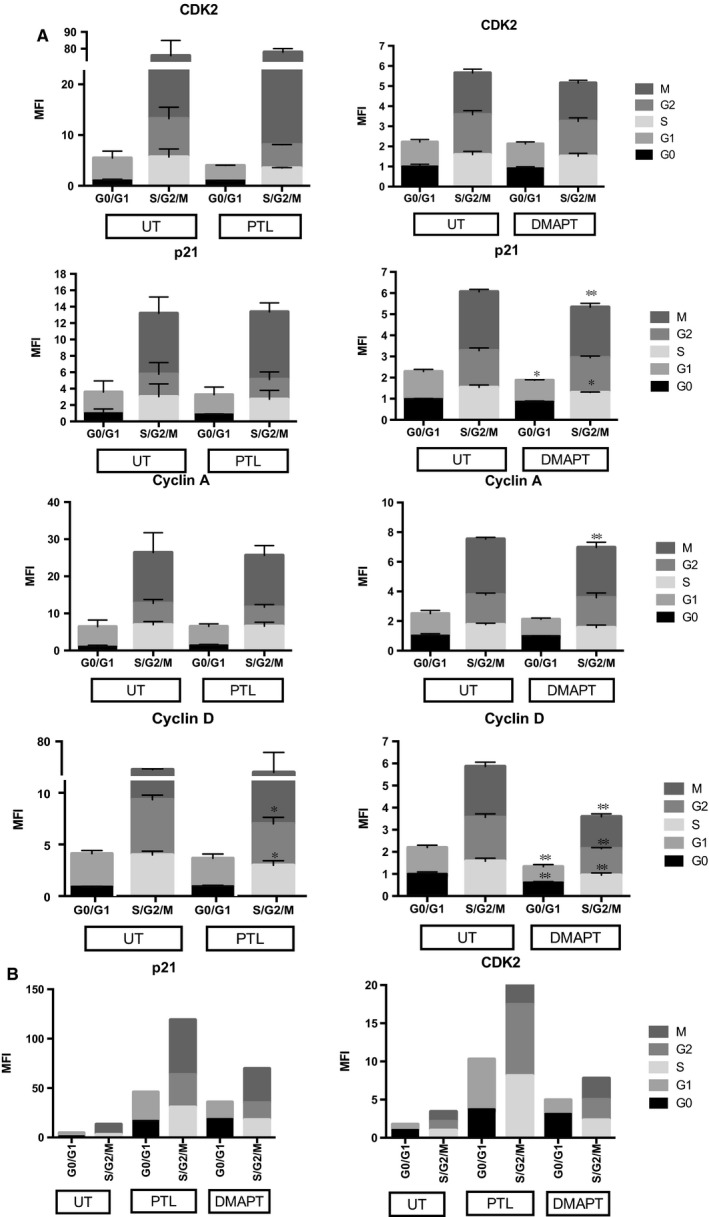
Effect of PTL and DMAPT in cell cycle regulators. A, K562 cell line was cultured by 48 h with 10 μmol/L of PTL or DMAPT and stained with specific conjugate antibodies to detect cell cycle regulators (CDK2, p21, D and A Cyclin) by flow cytometry. Results are expressed as Mean Fluorescent Intensity and represent the average of tree‐independent experiments MFI values where normalized to levels in UT G0 (considered as 1). B, shows the effect of PTL and DMAPT in CDK2 and P21 expression in cells CD34^+^lin^−^ from a chronic phase CML primary sample. Significance between UT and PTL or DMAPT was determines using an unpaired Student *t*‐test, *P < .05 **P < .01

To further evaluate the mechanism behind the cell cycle arrest in CML cells treated with PTL or DMAPT, we looked into protein levels of cell cycle regulators. K562 cells and CD34^+^lin^−^ cells from a CML‐CP patient were cultured, with or without 10 μmol/L PTL or DMAPT, for 48 hours. After treatment cells were stained for Cyclin D and A, p21, CDK2; also with 7AAD and anti‐Ki67, to determine cell cycle inhibitor changes within cell cycle stages. Surviving K562 cells showed down‐regulation of Cyclin A and D, and CDK2. To our surprise, we observed inhibition of p21 in K562 cells treated with DMAPT; this effect was not observed in CML CD34+ cells. Indeed, such cells showed a strong up‐regulation of this inhibitor with PTL and DMAPT treatment (Figure 7), suggesting an important role for p21 in the survival of CML primitive populations. It is noteworthy that a previous study in adult hematopoietic stem cells by Cheng and colleagues has linked p21 to quiescence.^28^

## 4 DISCUSSION

The use of plants containing sesquiterpene lactones has been part of folk medicine for centuries and across multiple cultures. This is in part due to the widespread of the Asteraceae family across the American and European territories. This has put an evolutionary pressure for members of this plant family to evolve different phytochemical mechanisms to evade predators, competitors and other protective mechanisms, making Asteraceae plants a rich reservoir of bioactive compounds.^29^

Recently, an interest has grown on the ability of parthenolide and its analogue DMAPT, to target malignant populations. Several reports have described effect on neoplastic cells from colorectal,^30^ lung and bladder cancer.^31^ Of particular importance is their reported capacity to target cancer stem cells, such as acute leukaemia^10^ and breast cancer stem cells.^32^ The knowledge that different cancers depend on a stem cell‐like population to maintain the disease which are resistant to therapy, thus causing relapse and or metastasis, has made it a priority in cancer research to find methodologies to target this population to find better treatments.

The overall data from this work indicate a robust effect of PTL and DMAPT on CML cells. CML cells exposed to PTL and DMAPT loss 50% viability by 24 hours, at a concentration around 7 μmol/L. The effect being more significant in cells from patients in CML‐CP than in CML cell lines (which are biologically more similar to CML‐BC cells), while CML‐CP cells with the same treatment showed viability to be around 30%. This different sensitivity between different progression phases of the disease would be consistent with the notion that leukaemic cells in blast crisis have acquired additional mutations^33^ that could make them more resistant to cell death mechanisms such as apoptotic induction by ROS.

We observed an increase in cell number when NBM MNC was treated with low concentrations of PTL and DMAPT. This effect, has been previously reported in studies where hematopoietic cells were treated with PTL.^17^ On the other hand Tothova and colleagues^34^ have reported an increased cell cycling and myeloid expansion associated with an increase in ROS. Hence, we could suspect a ROS‐dependent induction of proliferation. However, we did not observe a loss of CFSE fluorescence in cells treated with 2 μmol/L of sesquiterpene lactones, compared to untreated cells, which would suggest an increase in proliferation. Thus, the possible role of PTL, at low concentrations, in proliferation of normal cells needs to be further addressed.

We also found that the induction of cell death depended on ROS increase. Indeed, the ROS scavenger NAC was able to rescue cells from death and this correlated with absence of ROS induction. Interestingly, this effect was observed in CML CD34^+^lin^−^ cells, but not on their normal counterpart. NBM primitive cells showed lower basal levels of ROS than their CML counterpart, this could indicate higher levels of reduced glutathione in normal populations, which would make normal primitive cells resilient to PTL and DMAPT effect as has been observed in CD34+ cells from AML samples,^13^ and proves a key mechanism to be exploited and further studied. PTL and DMAPT also inhibited phosphorylation and nuclear localization of NF‐κB (p65). Interestingly, CML CD34^+^lin^−^ cells from chronic phase showed basal phosphorylation and nuclear localization of p65, indicating a basal activity of this transcription factor in the primitive population, suggesting that the NF‐κB pathway has biological importance in the CML stem/progenitor compartment, which would be in keeping with the fact that NF‐κB inhibition in leukaemia cells has been described as a key factor in achieving LSC eradication in AML by Guzman and colleagues.^35^ Nevertheless, on the basis of our results, we propose that the most important mechanism of PTL and DMAPT effect on CML cells (without affecting normal cells in a significant manner) is induction of ROS.

One of the hallmarks of CML, is the increase in cell proliferation.^36^ TKIs, which directly target the activation of Bcr‐Abl, inhibit proliferation of bulk CML cells; however, a fraction of LSC has been shown to enter quiescence and avoid apoptosis.^15^ Here, we found that a fraction of CML cells is able to survive PTL and DMAPT treatment. This seems to be linked to cell cycle arrest, which could be dependent on NF‐κB inhibition. Interestingly, ROS levels have also been related to cell proliferation. Indeed, in hematopoietic cells they have been linked to FoxO transcription factors, which play an important role in myeloid differentiation. Deletion of FoxO transcription factors in HSC results in increase in ROS levels and differentiation to the myeloid lineage.^34^ With this in mind, we expected to find the arrest on cell cycle to be reversed by NAC pre‐treatment; however, to our surprise we found that while cells were consistently rescued from cell death with NAC pre‐treatment, they readily suffered a cell cycle arrest that could not be explained by ROS induction.

NF‐κB activity in CML has been previously identified as a critical pathway, particularly in imatinib resistant cell lines,^37^ and suggested as a therapeutic target. Since we found cell cycle changes to be independent of ROS induction, we sought to further address the mechanism behind cell cycle arrest after sesquiterpene lactone treatment. Thus, we looked into protein levels of cell cycle regulators. Surviving cells from K562 cell line and CML CD34^+^lin^−^ cells after 48 hours treatment showed changes in cyclin D1 and A, p21, and CDK2. K562 cells showed down‐regulation of Cyclin D, which is critical in the G1 to S transition. This effect would be consistent with the inhibition of NF‐κB as has been reported by Hinz and colleagues,^38^ also it is the strongest known link between NF‐κB pathway and cell cycle, as mentioned by Joyce and collaborators.^39^ We also found a decrease tendency in CDK2 and Cyclin A, which is mostly linked to G2/S cell cycle progression, which would suggest an involvement with the K562 arrest observed in the G2 phase. On the other hand, CD34^+^lin^−^ cells from a CML patient showed a marked induction of p21 inhibitor and an increase in CDK2. Interestingly, we did not observe a G2 cell cycle arrest or an S phase burden in this cell population; instead, a cell cycle arrest was strongly observed in the G0/G1 compartment. The absence of a G2 arrest could be explained by an up‐regulation of CDK2, however further assessment would be necessary to understand the mechanism behind the cell cycle arrest induced by sesquiterpene lactone.

Taken together, our data show a strong inhibitory effect of PTL and DMAPT on bulk CML cells and on a more primitive CD34^+^lin^−^ cell population. We also observed that PTL‐induced cell death is accompanied by cell cycle arrest in surviving cells. Our findings indicate that cell cycle arrest we observed after PTL and DMAPT treatment is ROS independent, and changes in cell cycle regulators would be consistent with NF‐κB inhibition. We speculate that evading cell cycle arrest could increase and potentiate PTL and DMAPT effect on the CML model. Finally, it is important to comment that it is necessary to consider an in vivo approach to consider PTL and DMAPT as selective molecules to eliminate CML primitive cells.

## CONFLICTS OF INTEREST

The authors confirm that there are no conflicts of interest.
